# Supplementary material for: SARS-CoV-2 infection establishes a stable and age-independent CD8+ T cell response against a dominant nucleocapsid epitope using restricted T cell receptors
Source: Nat Commun. 2023 Oct 23;14:6725. doi: 10.1038/s41467-023-42430-z (PMC10593757; doi:10.1038/s41467-023-42430-z)
Supplement: Supplementary file 1 — Supplementary Information [file 41467_2023_42430_MOESM1_ESM.pdf]

SARS-CoV-2 infection establishes a stable and age-independent CD8$^+$ T cell response against a dominant nucleocapsid epitope using restricted T cell receptors

Authors:
Cecily Choy[1*], Joseph Chen[1*], Jiangyuan Li[1], D. Travis Gallagher[2], Jian Lu[1], Daichao Wu[3], Ainslee Zou[1], Humza Hemani[1], Beverly A. Baptiste[1], Emily Wichmann[1], Qian Yang[1], Jeffrey Ciffelo[1], Rui Yin[3], Julia McKelvy[4], Denise Melvin[4], Tonya Wallace[1], Christopher Dunn[1], Cuong Nguyen[1], Chee W. Chia[4], Jinshui Fan[5], Jeannie Ruffolo[6], Linda Zukley[6], Guixin Shi[7], Tomokazu Amano[8], Yang An[9], Osorio Meirelles[10], Wells W. Wu[11], Chao-Kai Chou[11], Rong-Fong Shen[11], Richard A. Willis[12], Minoru S.H. Ko[8], Yu-Tsueng Liu[7], Supriyo De[5], Brian G. Pierce[3], Luigi Ferrucci[6], Josephine Egan[4], Roy Mariuzza[3], and Nan-ping Weng[1*#]

Affiliations:
[1] Laboratory of Molecular Biology and Immunology, National Institute on Aging, NIH, Baltimore, MD, USA
[2] National Institute of Standards and Technology (NIST), Gaithersburg, MD, USA
[3] W.M. Keck Laboratory for Structural Biology, University of Maryland Institute for Bioscience and Biotechnology Research, Rockville, MD, USA
[4] Laboratory of Clinical Investigation, National Institute on Aging, NIH, Baltimore, MD, USA
[5] Computational Biology and Genomics Core, Laboratory of Genetics and Genomics, National Institute on Aging, NIH, Baltimore, MD, USA
[6] Translational Gerontology Branch, National Institute on Aging, NIH, Baltimore, MD, USA
[7] Diagnologix LLC, San Diego, CA, USA
[8] Elixirgen Therapeutics, Inc. MD, USA
[9] Laboratory of Behavioral Neuroscience, National Institute on Aging, NIH, Baltimore, MD, USA
[10] Laboratory of Epidemiology & Population Sciences, National Institute on Aging, NIH, Baltimore, MD, USA
[11] Facility for Biotechnology Resources, CBER, Food and Drug Administration, Silver Spring, MD, USA
[12] NIH Tetramer Core Facility at Emory University, Atlanta, GA, USA

[*] These authors contributed equally.
[#] Correspondence to Nan-ping Weng, Wengn@nih.gov

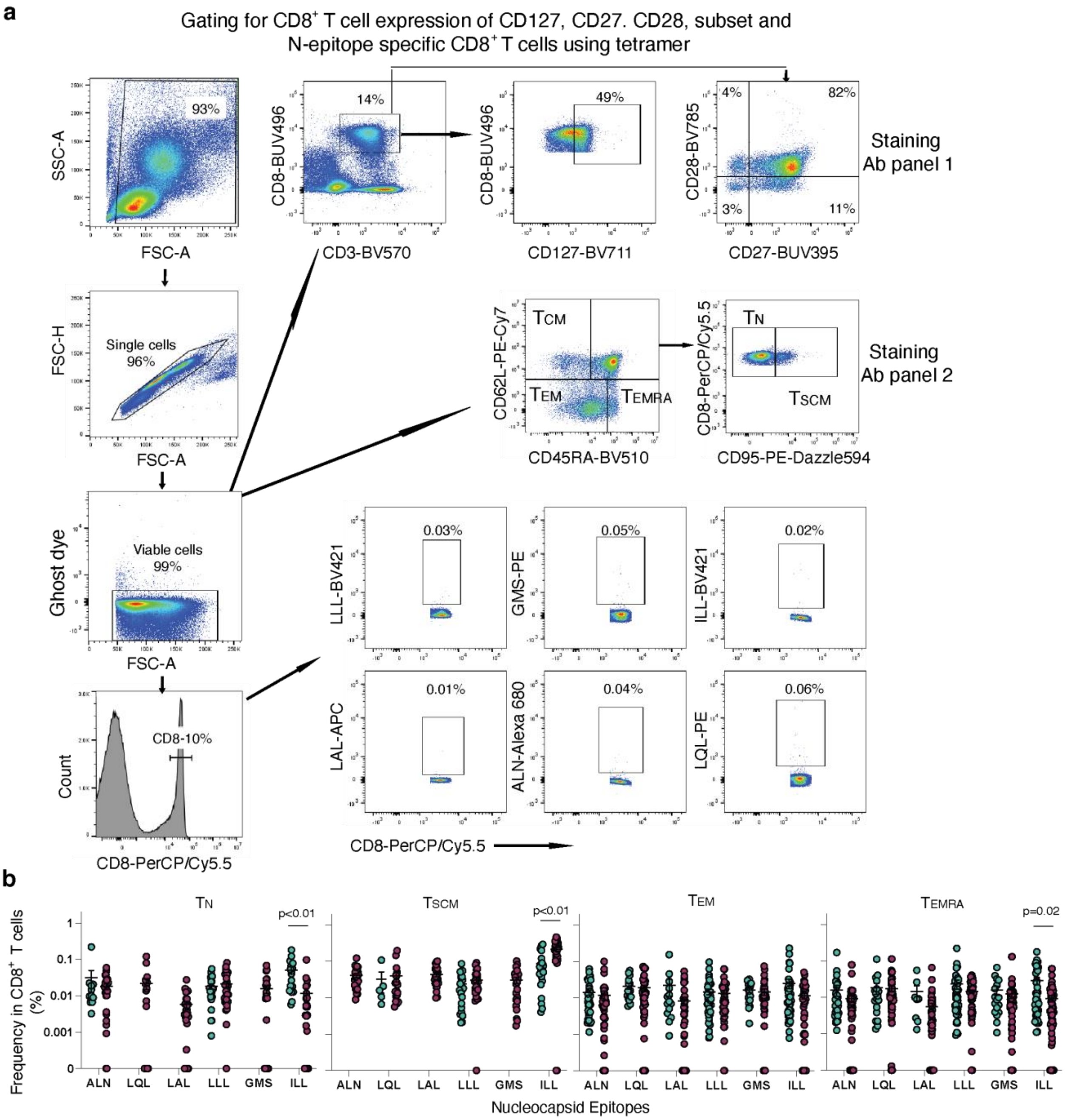

Supplementary Figure 1. Gating strategies for flow cytometry analysis of CD8$^+$ T cells and CD8$^+$ T cell subsets specific for six SARS-CoV-2 nucleocapsid epitopes. a. Gating strategies for CD8$^+$ T cell and its five subsets: Naïve (T_N), memory stem cell (T_{SCM}), central (T_{CM}) and effector (T_{EM}) memory cells, and effector memory cells expressing CD45RA (T_{EMRA}) for six nucleocapsid epitopes by stained by the specific tetramers, and for CD127, CD27 and CD28. **b**. Frequencies of four subsets of CD8$^+$ T cells that recognizes six nucleocapsid epitopes in convalescence and uninfected donors (Convalescent=35, Uninfected=80). Two-tailed T-test adjusted for age and sex was carried out for all comparisons between convalescent and uninfected donor. p value, mean and SEM are shown.

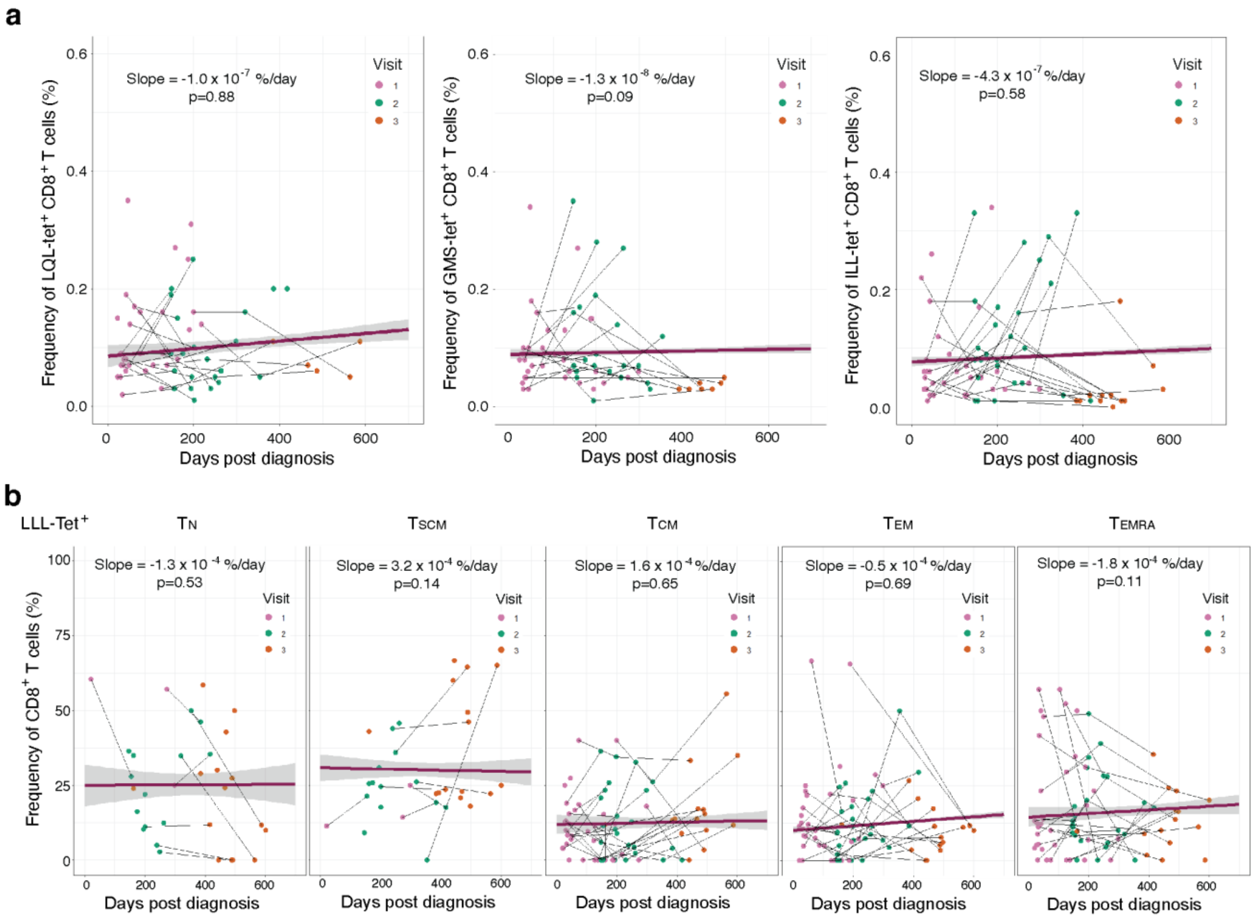

Supplementary Figure 2. Frequencies of CD8$^+$ T cells recognizing N nucleocapsid change over time in convalescent patients. **a**. Frequency of tetramer positive (LQL, GMS, and ILL) CD8$^+$ T cells at multiple time points (pink=visit 1, green=visit 2, orange=visit 3). Multiple visits for a single subject are connected by thin black lines. The overall trend line (purple) across time points for each subject were generated by the mixed effect linear regression model (adjusted for age and sex) with error bounds indicating a 95% confidence interval (light grey) for the convalescent cohort, n=75. **b**. Frequencies of LLL-tetramer positive CD8$^+$ T cell subsets over time.

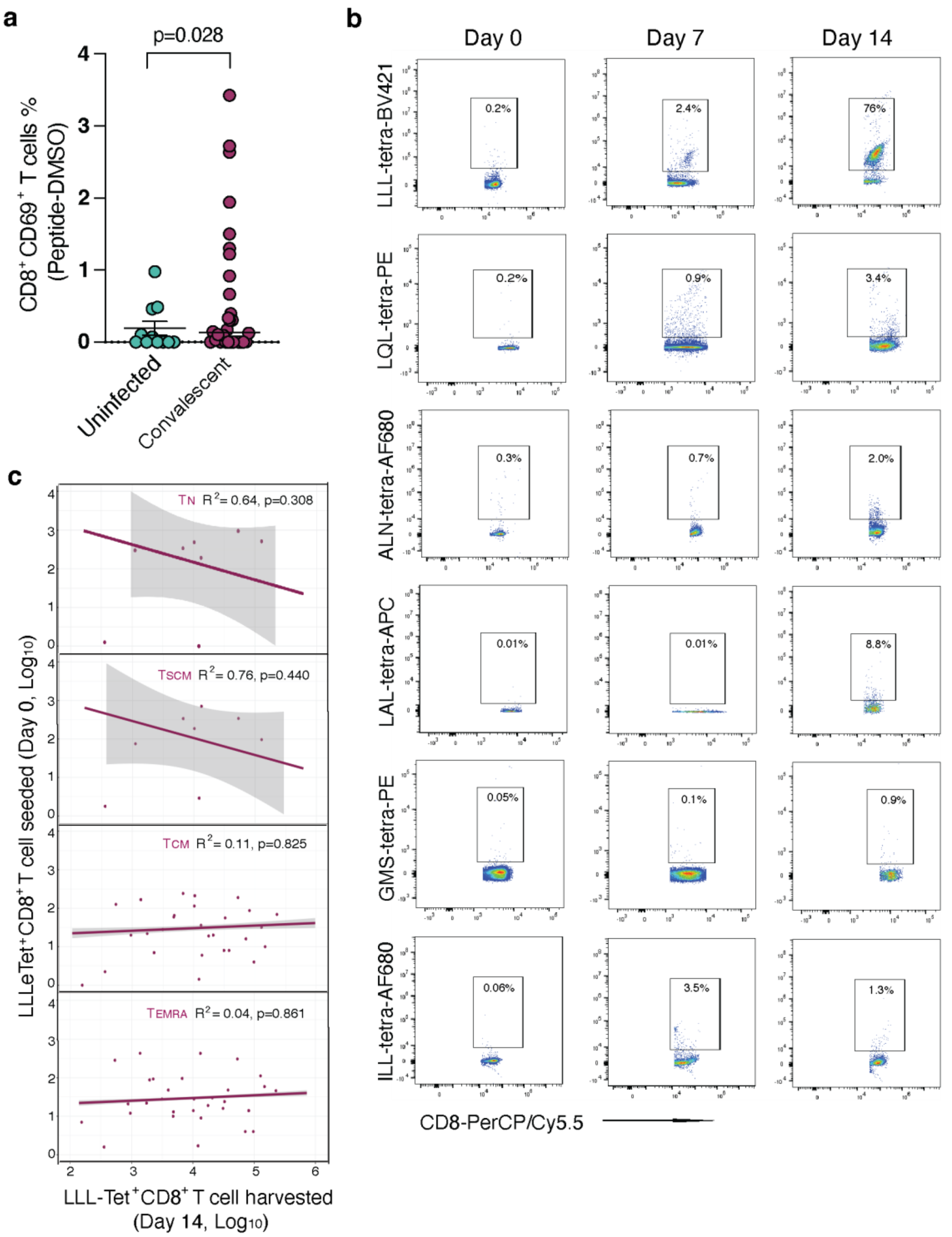

Supplementary Figure 3. Functional assessment of the ability for *in vitro* expansion of SARS-CoV-2 nucleocapsid-specific CD8$^+$ T cells. **a**. Flow cytometry analysis of CD8$^+$CD69$^+$ T cells after peptide stimulation overnight. Convalescent patients=30 and uninfected donor=11. p value, mean and SEM are shown. **b**. Representative flow gating of six nucleocapsid specific CD8$^+$ T cells prior (Day 0) and post (Day 7 and 14) *in vitro* peptide stimulation. **c**. Correlation between seeding LLL-Tet$^+$ CD8$^+$ T cell subset counts on day 0 and harvested LLL-Tet$^+$ CD8$^+$ T cell counts after stimulation. Values were $\log_{10}$ transformed and a linear regression was generated comparing the correlations of day 0 and day 14 with error bounds indicating a 95% confidence interval (light grey). Two-tailed T-test adjusted for age and sex was carried out for all comparisons between convalescent and uninfected donor. p value, R^2 value, and SEM are shown.

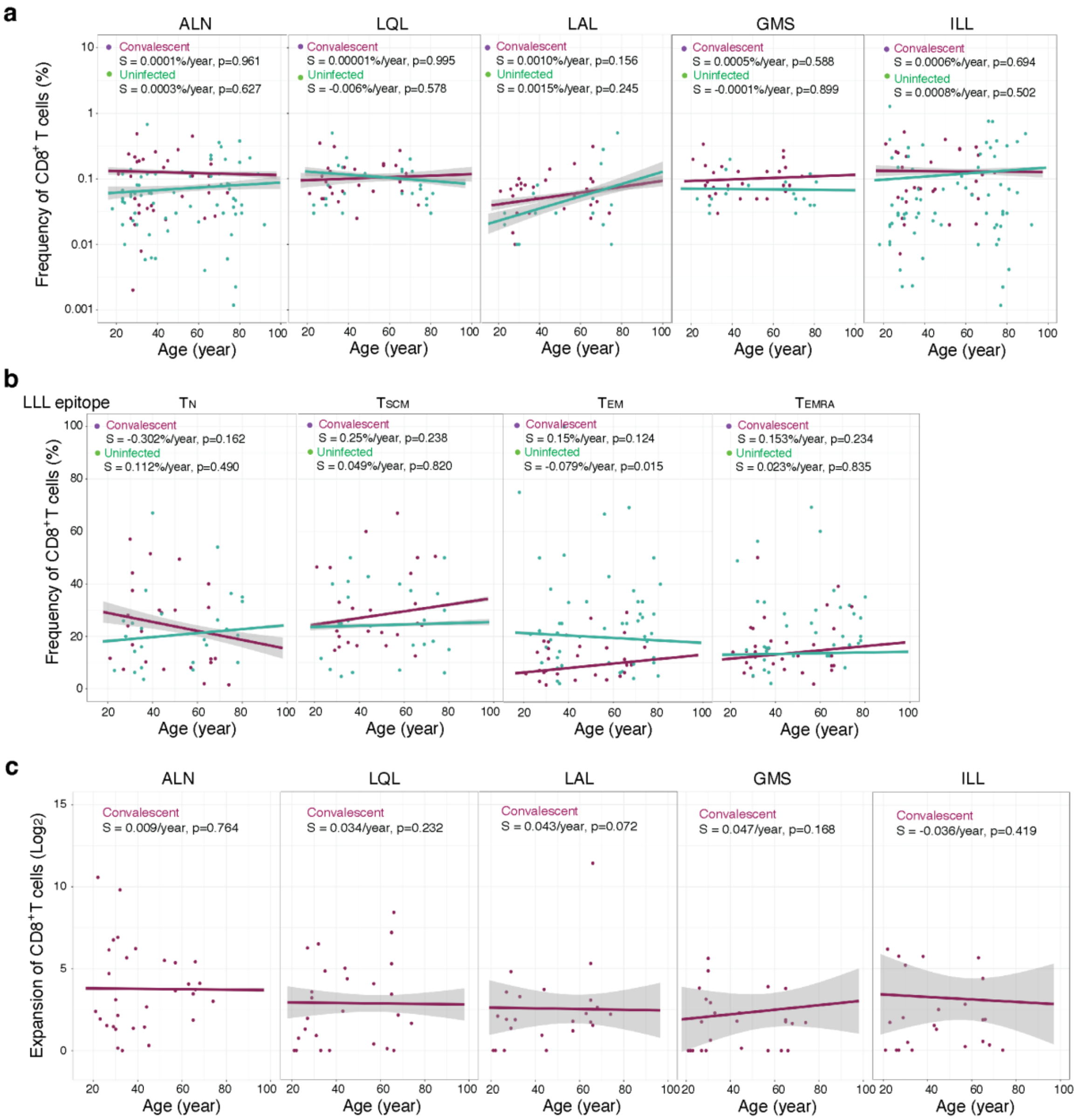

Supplementary Figure 4. Age-associated changes in nucleocapsid specific CD8$^+$ T cells in uninfected and convalescent donor. **a**. Correlation between frequency of five nucleocapsid epitope-specific CD8$^+$ T cells and donor's age. For donors with multiple visits, the frequencies were averaged and depicted as a single point. Purple line represents convalescent donors and green line represent uninfected donors, and they were generated using a linear regression model adjusted with sex with error bounds indicating a 95% confidence interval (light grey) (Convalescent=34, Uninfected=57). **b.** Correlation between frequency of LLL-Tet$^+$ CD8$^+$ T cell subsets and donor's age. For donors with multiple visits, the frequencies were averaged and depicted as a single point. Purple line represents convalescent donors and green line represent uninfected donors, and they were generated using a linear regression model adjusted with sex with error bounds indicating a 95% confidence interval (light grey) (Convalescent=34, Uninfected=57). **c**. Correlation between degree of nucleocapsid-specific CD8$^+$ T cell expansion and convalescent donor's age (n=35).

a Experimental design of confirming LLL-Tet+ TCR specificity

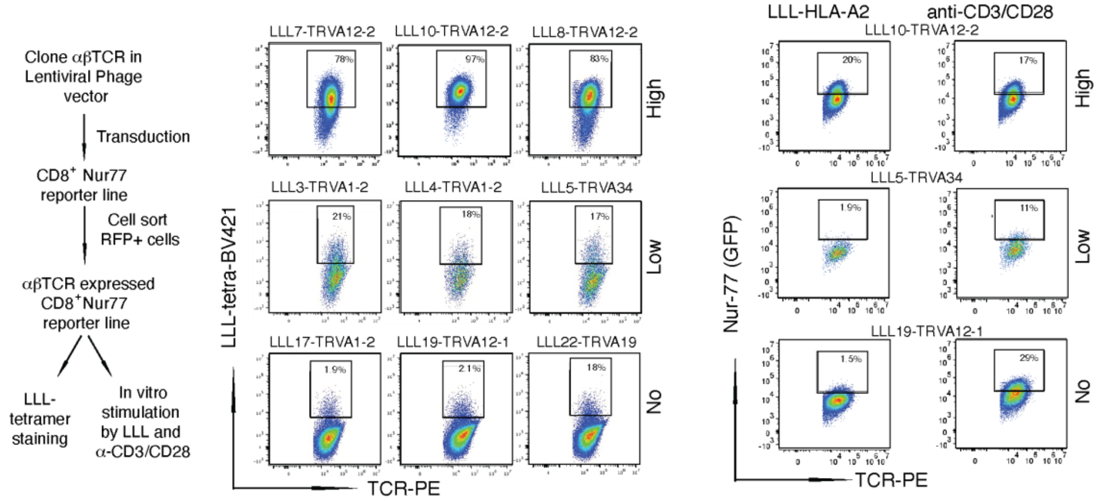

b CDR3 motif pairing of three LLL-TCR clusters

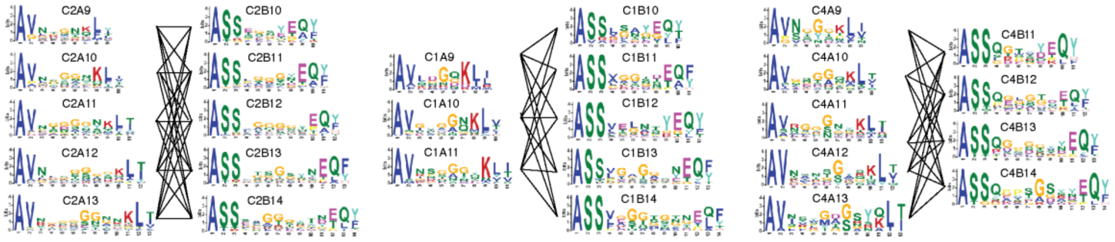

	C2B10	C2B11	C2B12	C2B13	C2B14	Total
C2A9	1.2%	0.6%	0.6%	0.4%	0.4%	3.1%
C2A10	0.8%	1.7%	1.2%	1.0%	0.2%	4.8%
C2A11		1.4%	1.2%	1.2%	0.8%	4.5%
C2A12	0.4%	0.8%	0.8%	1.7%	0.4%	4.1%
C2A13	0.2%	1.0%	2.3%	1.4%	0.4%	5.2%
Total	2.5%	5.4%	6.0%	5.6%	2.1%	21.7%

	C1B10	C1B11	C1B12	C1B13	C1B14	Total
C1A9	0.2%		1.2%	1.0%	0.2%	2.5%
C1A10	0.6%	0.6%	2.9%	1.0%	0.2%	5.2%
C1A11	0.2%	0.4%	0.2%	0.8%	0.4%	1.9%
Total	1.0%	1.0%	4.3%	2.7%	0.8%	9.7%

	C4B11	C4B12	C4B13	C4B14	Total
C4A9		0.6%	0.2%	0.4%	1.2%
C4A10	0.2%	0.8%	0.8%	0.6%	2.3%
C4A11	0.4%	0.4%	1.0%	0.2%	1.9%
C4A12	0.6%		0.8%	0.6%	1.9%
C4A13	0.2%		0.4%	0.2%	0.8%
Total	1.4%	1.7%	3.1%	1.9%	8.1%

c Diagram of machine learning RF model

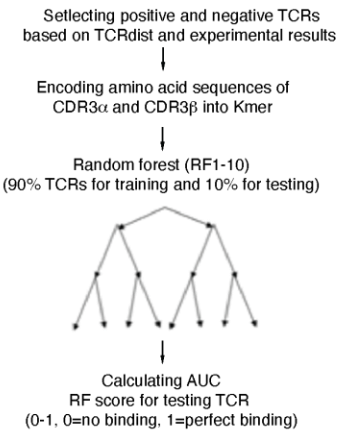

d Top most influenced kmers

Chain	kmer	Location	Contribution	Model used
alpha	AVN	L-End	0.76%	7
alpha	AVK	L-End	0.73%	2
alpha	KLI	R-End	0.49%	1
alpha	NYQ	Center	0.32%	3
alpha	GYS	Center	0.24%	4
beta	ASS	L-End	0.68%	5
beta	TQY	R-End	0.49%	1
beta	EQY	R-End	0.40%	3
beta	GQG	Center	0.36%	2
beta	SSG	L-Side	0.24%	4

Supplementary Figure 5. Analysis of LLL+ specific TCRs. A. Experimental design for confirmation of LLL-binding specificity. a Diagram of experimental steps for introducing TCR to NJ76-CD8 (Nur77 reporter) cells. Representative flow graphs of high, low and no tetramer bindings, as well as of GFP reporter activity after 4-hour stimulation with either LLL-HLA-A2/antiCD28 or antiCD3/antiCD28. **b**. CDR3 motifs of LLL+ TCRs of three clusters. **c**. Diagram of machine learning steps. **d**. Information of top 5 kmers with highest perturbation score that has most influenced on RF score (presented as percentage) and number of models displayed perturbation.

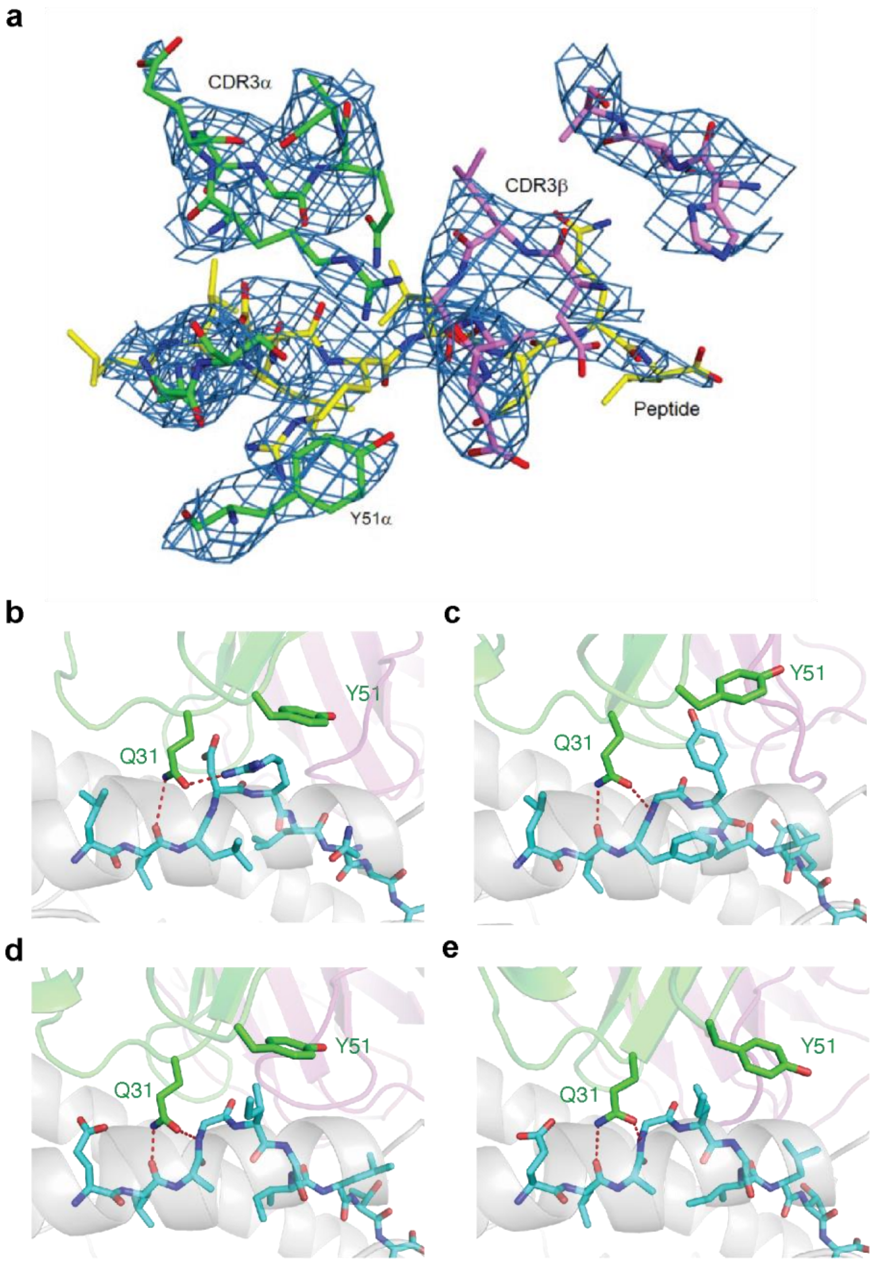

Supplementary Figure 6. Electron density in the interface of the LLL8–LLL–HLA-A2 complex and shared pMHC engagement by TRAV12-2 TCRs. a. Density from the final $2F_o - F_c$ map at 3.18 Å resolution is contoured at 1.2 s. TCRs: **b**. LLL8, **c.** A6, **d**. DMF5, and **e**. Mel5 are shown in complex with respective target peptide and HLA-A2 MHC. A6, DMF5, and Mel5 TCR–pMHC complexes are from PDB entries 1AO7, 3QDG, and 3HG1, respectively. All complexes are shown in a common reference frame based on MHC helices, with MHC shown as gray cartoon, peptide as cyan sticks, TCR a chain as green cartoon, and TCR β chain as magenta cartoon. LLL8 residues Q31a and Y51a and equivalent residues in other TCRs are shown as sticks, all labeled by LLL8 TCR numbering for clarity. Dashed red lines denote hydrogen bonds between Q31a and peptide backbone atoms, with additional Q31a–peptide side chain hydrogen bond shown for TCR LLL8.

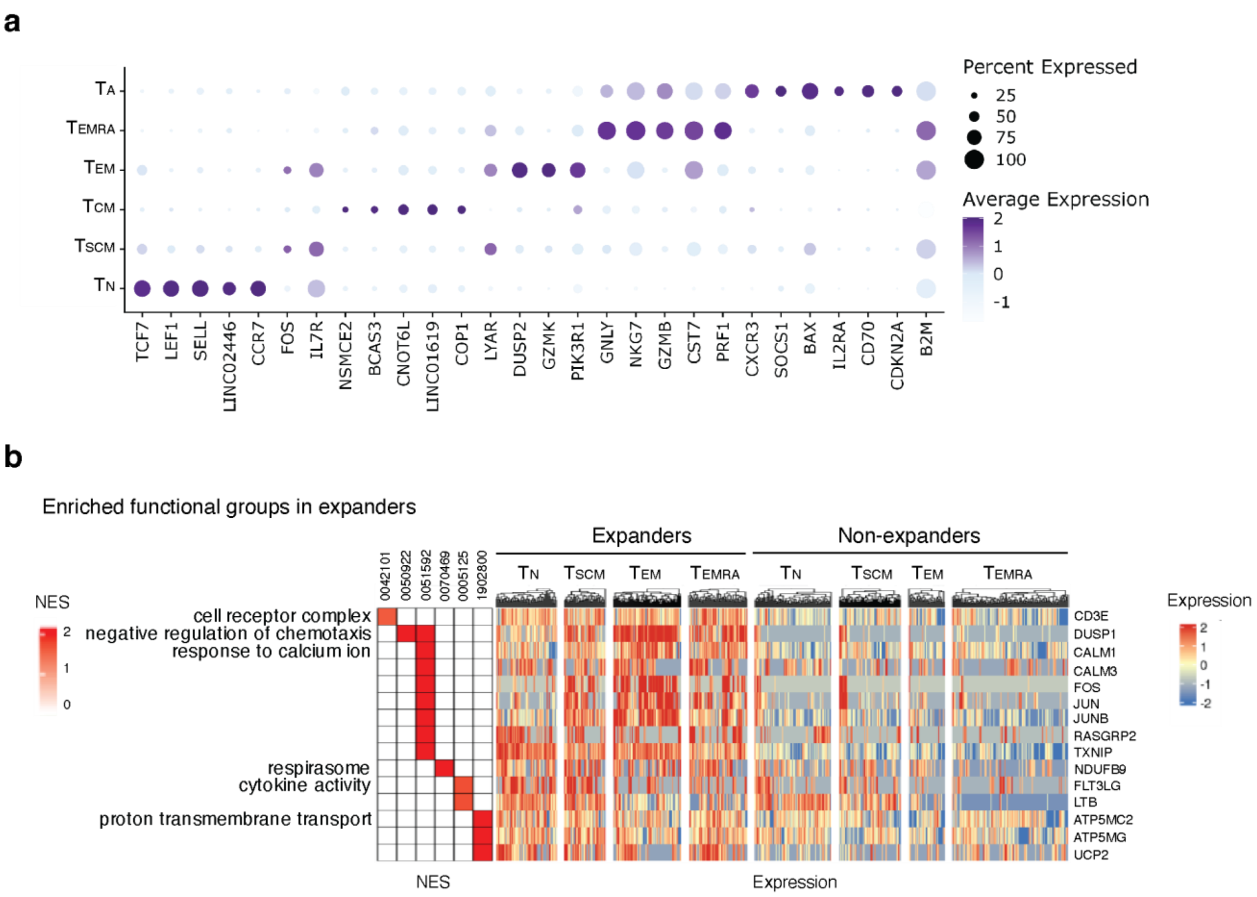

Supplementary Figure 7. Differentially expressed genes that define the six subsets of CD8$^+$ T cells and enriched functional groups of genes in CD8$^+$ T cells from the expanders. a. Differentially expressed genes in each of six subsets of CD8$^+$ T cells. **b**. Enriched expressed genes in CD8$^+$ T cells and subsets of expanders heatmap of selected enriched genes of freshly isolated CD8$^+$ T cells of expanders compared to non-expanders. NES=Normalized enrichment score.

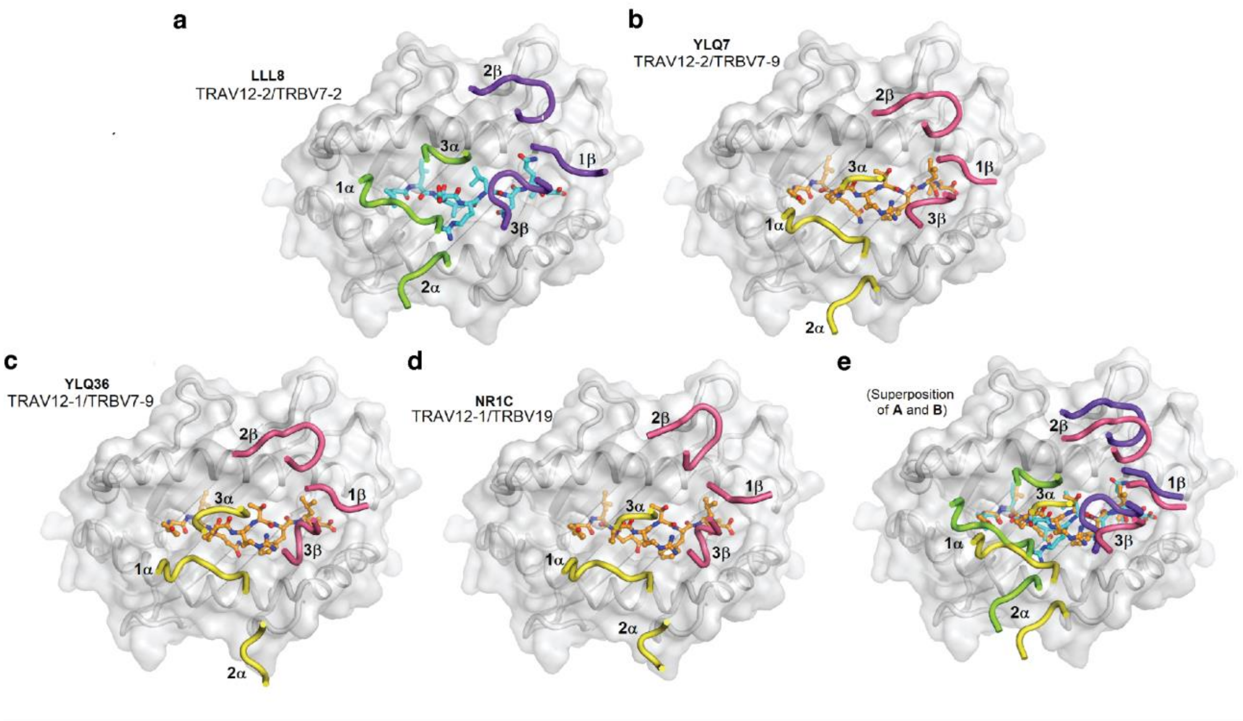

Supplementary Figure 8. Comparison of docking topologies of SARS-CoV-2-specific TCRs. Top views of TCR–pMHC complexes featuring SARS-CoV-2 epitopes presented by HLA-A2 and TCRs that use germline genes TRAV12-1 and TRAV12-2. **a.** The LLL8–LLL–HLA-A2 complex, with the LLL epitope from the nucleocapsid protein shown in cyan and the CDR loops in green and purple. The gene usage of this TCR is given in the inset, TRAV12-2 and TRBV7-2. **b**. The YLQ7–YLQ–HLA-A2 complex (PDB code 7N1F). Gene usage: TRAV12-2/TRBV7-9. The spike epitope YLQ is colored orange, with the CDRs in yellow and pink. **c**. The YLQ36–YLQ–HLA-A2 complex (7PBE). Gene usage: TRAV12-1/TRBV7-9. **d**. The NR1C–YLQ–HLA-A2 complex (7N6E). Gene usage: TRAV12-1/TRBV19. **e**. Superposition of LLL8–LLL–HLA-A2 and YLQ7–YLQ–HLA-A2 for comparison of docking topologies.

Supplementary Table 1. Comparison of six N protein epitopes between SARS-CoV-2 and four common cold coronaviruses

virus	ALN	
	sequence[a]	SARS-CoV-2 match[b]
SARS-CoV-2	ALNTPKDHI	9
OC43	DVNTPADIV	4
HKU1	DTSTPSDVS	3
NL63	AKTVNTSLG	1
229E	AKTEPTGYG	2
	LQL	
SARS-CoV-2	LQLPQGTTL	9
OC43	TRFPPGTVL	4
HKU1	TRFPPGTIL	4
NL63	LEPKFSIAL	2
229E	EIPHFNQKL	1
	LLL	
SARS-CoV-2	LLLDRLNQL	9
OC43	LVLAKLGKD	3
HKU1	LVLAKLGKD	3
NL63	AVNLALKNL	2
229E	AVAAALKSL	2
	LAL	
SARS-CoV-2	LALLLLDRL	9
OC43	IASLVLAKL	4
HKU1	IANLVLAKL	4
NL63	LVAAVNLAL	2
229E	IMKAVAAAL	1
	GMS	
SARS-CoV-2	GMSRIGMEV	9
OC43	FGSKLELAK	1
HKU1	FGSKLDLVK	1
NL63	FDSEVSTEE	2
229E	FDSHIVSKE	2
	ILL	
SARS-CoV-2	ILLNKHIDA	9
OC43	KVLSENLNA	2
HKU1	KVLEENLNA	2
NL63	PKFIEQISA	2
229E	EELNAFTRE	2

[a]N protein sequences for coronaviruses OC43, HKU1, NL63, and 229E were obtained from NCBI Genbank (Accession QXL74890.1, YP_173242.1, ABK63972.1, and AGW80953.1) and aligned with SARS-CoV-2 N protein using MAFFT software[1]. Sequences shown represent residues aligned to SARS-CoV-2 epitope. In cases of gaps in epitope alignment, sequences were realigned using local pairwise ungapped alignment.
[b]Number of residues identical with SARS-CoV-2 epitope residues.

Reference

Supplementary Table 2: Summary of *in vitro* stimulated LLL-Tet^{+} CD8^{+} T cells

Category	Donor ID	Visit	Expansion (Log2)	LLL+ at D14	Degree of expansion
Convalescent	C006	2	5.32	0.4%	Mild Expansion
Convalescent	C009	2	5.34	0.6%	Robust expansion
Convalescent	C009	3	3.78	0.3%	Mild Expansion
Convalescent	C014	1	2.47	0.0%	Mild expansion
Convalescent	C014	2	7.08	1.1%	Robust Expansion
Convalescent	C014	3	3.97	0.2%	Mild Expansion
Convalescent	C016	2	4.73	0.2%	Mild Expansion
Convalescent	C016	3	10.84	2.5%	Robust Expansion
Convalescent+Vaccine	C020	2	7.80	0.9%	Robust Expansion
Convalescent+Vaccine	C020	3	3.85	0.9%	Robust expansion
Convalescent+Vaccine	C021	3	4.81	0.8%	Robust Expansion
Convalescent	C023	1	6.48	5.0%	Robust expansion
Convalescent	C024	1	3.62	2.1%	Robust expansion
Convalescent	C025	2	7.50	0.3%	Mild Expansion
Convalescent	C025	3	2.15	0.6%	Mild Expansion
Convalescent	C026	1	8.74	11.0%	Robust Expansion
Convalescent	C026	2	0.57	0.1%	No Expansion
Convalescent	C027	2	3.10	4.1%	Robust Expansion
Convalescent	C029	1	10.26	31.6%	Robust Expansion
Convalescent	C029	2	1.63	1.8%	Mild expansion
Convalescent	C029	3	7.56	1.9%	Robust Expansion
Convalescent+Vaccine	C030	1	9.21	18.2%	Robust Expansion
Convalescent+Vaccine	C030	2	2.36	0.3%	Mild Expansion
Convalescent+Vaccine	C030	3	11.06	8.8%	Robust Expansion
Convalescent	C031	1	9.41	10.9%	Robust Expansion
Convalescent	C031	2	3.99	0.5%	Mild Expansion
Convalescent	C034	1	8.38	7.9%	Robust expansion
Convalescent	C034	2	3.40	0.1%	Mild Expansion
Convalescent	C037	1	5.78	0.8%	Robust Expansion
Convalescent	C037	2	0.76	0.1%	No Expansion
Convalescent	C037	3	3.67	0.4%	Mild Expansion
Convalescent	C038	1	4.19	0.9%	Robust Expansion
Convalescent	C038	2	0.00	0.1%	No Expansion
Convalescent	C039	1	7.21	3.2%	Robust Expansion
Convalescent	C039	2	8.82	5.6%	Robust Expansion
Convalescent+Vaccine	C040	1	6.94	4.8%	Robust Expansion
Convalescent	C040	2	5.88	7.5%	Robust Expansion
Convalescent	C042	1	8.44	4.4%	Robust Expansion
Convalescent	C042	2	12.47	71.3%	Robust Expansion
Convalescent	C042	3	9.72	2.9%	Robust Expansion
Convalescent	C043	1	2.61	0.1%	Mild expansion
Convalescent	C044	1	7.21	0.3%	Mild Expansion
Convalescent	C045	1	3.82	0.2%	Mild Expansion
Convalescent+Vaccine	C046	1	11.62	44.7%	Robust Expansion
Convalescent+Vaccine	C046	2	4.89	18.6%	Robust Expansion
Convalescent	C052	1	2.57	0.6%	Mild Expansion
Convalescent	C052	2	0.99	0.4%	No Expansion
Convalescent	C052	3	5.77	0.3%	Robust expansion
Convalescent	C054	1	4.35	0.2%	Robust expansion
Convalescent	C054	2	0.00	0.3%	No expansion
Convalescent	C055	1	0.00	0.0%	No expansion
Convalescent	C062	1	6.96	4.8%	Robust Expansion
Convalescent+Vaccine	C063	1	1.31	0.4%	Mild expansion
Convalescent	C063	2	0.00	0.0%	No expansion
Convalescent	C066	1	10.11	20.8%	Robust Expansion

Convalescent+Vaccine	C070	1	0.78	0.9%	No Expansion
Convalescent+Vaccine	C070	3	4.97	1.0%	Robust expansion
Convalescent	C072	1	0.00	0.1%	No Expansion
Convalescent	C074	1	1.82	0.3%	Mild expansion
Convalescent	C074	2	2.55	0.2%	Mild expansion
Convalescent	C074	3	4.04	0.8%	Robust Expansion
Convalescent	C079	1	7.23	18.4%	Robust Expansion
Convalescent	C079	2	8.57	4.0%	Robust Expansion
Convalescent	C080	1	2.31	0.8%	Mild Expansion
Convalescent	C081	1	1.09	0.4%	Mild Expansion
Convalescent	C081	2	10.86	19.8%	Robust Expansion
Uninfected	U001	1	1.03	0.5%	Mild Expansion
Uninfected	U002	1	0.20	0.3%	No Expansion
Uninfected	U003	1	0.16	0.5%	No Expansion
Uninfected	U006	1	2.37	0.0%	Mild expansion
Uninfected	U019	1	12.47	71.3%	Robust expansion
Uninfected	U029	1	0.00	0.1%	No expansion
Uninfected	U034	1	2.89	0.0%	Mild Expansion
Uninfected	U042	1	0.00	0.2%	No Expansion
Uninfected	U047	1	0.00	0.1%	No Expansion
Uninfected	U048	1	0.00	0.1%	No Expansion
Uninfected	U049	1	0.00	0.2%	No Expansion
Uninfected	U051	1	0.00	0.1%	No expansion
Uninfected	U055	1	0.00	0.1%	No Expansion
Uninfected	U056	1	5.98	0.1%	Mild Expansion
Uninfected	U057	1	2.25	0.2%	Mild expansion
Uninfected	U058	1	7.02	0.2%	Mild Expansion
Uninfected	U060	1	6.95	0.6%	Robust expansion
Uninfected	U063	1	2.27	0.0%	Mild expansion
Uninfected	U065	1	7.08	1.1%	Robust expansion
Uninfected	U066	1	5.00	0.2%	Mild Expansion
Uninfected	U072	1	3.05	0.2%	Mild Expansion
Uninfected	U073	1	0.25	0.2%	No expansion
Uninfected	U074	1	5.82	7.2%	Robust expansion
Uninfected	U075	1	0.00	0.1%	No expansion
Uninfected	U090	1	0.00	0.7%	No expansion
Uninfected	U094	1	2.89	0.0%	Mild expansion
Uninfected	U095	1	1.69	0.1%	Mild expansion
Uninfected	U119	1	2.87	0.4%	Mild Expansion
Uninfected	U120	1	4.79	0.5%	Robust expansion
Uninfected	U121	1	3.22	0.8%	Robust expansion
Uninfected	U122	1	3.21	1.0%	Robust Expansion
Uninfected	U124	1	1.59	0.8%	Mild Expansion
Uninfected	U126	1	3.66	0.2%	Mild Expansion
Uninfected	U128	1	0.00	0.1%	No Expansion
Uninfected	U129	1	0.00	0.1%	No Expansion
Uninfected	U130	1	0.00	0.3%	No Expansion
Uninfected	U131	1	0.00	0.0%	No Expansion
Uninfected	U132	1	0.00	0.2%	No expansion
Uninfected	U134	1	0.00	0.2%	No Expansion
Uninfected	U135	1	0.00	0.0%	No Expansion
Uninfected	U136	1	0.00	0.1%	No Expansion
Uninfected	U137	1	2.69	0.2%	Mild Expansion
Uninfected	U138	1	0.00	0.3%	No Expansion

Supplementary Table 3. Data collection and refinement statistics

PDB accession code	8DNT
**Data collection**	
Resolution range (Å)[a]	48.7–3.11 (3.19–3.11)
Space group	C121
Unit cell parameters	182.2 Å, 121.8 Å, 210.6 Å<br>90°, 100.0°, 90°
Total reflections[a]	163,220 (4,296)
Unique reflections[a]	64,385 (2,708)
Multiplicity	2.5 (1.6)
Completeness (%)[a]	79.2 (45.5)
Mean $I/\sigma(I)$	8.0 (0.4)
Wilson B factor (Å^2)	107.6
R_{merge}[a,b]	0.097 (1.381)
$CC_{1/2}$[a]	0.997 (0.249)
**Refinement**	
Resolution range (Å)	30.0–3.18 (3.26–3.18)
Reflections used in refinement[a]	54,229 (2,345)
R_{work}[a,c]	0.221 (0.344)
R_{free}[a,c]	0.313 (0.431)
No. of protein atoms	26,210
No. of waters	0
Protein residues	3,300
r.m.s.d. from ideality	
Bond lengths (Å)	0.002
Bond angles (°)	0.831
Ramachandran plot statistics	
Favored (%)	85.3
Allowed (%)	12.5
Disallowed (%)	2.2
Rotamer outliers (%)	9.1
Clashscore	10.0
Average B factor (Å^2)	155.0

[a]Values in parentheses correspond to the highest resolution shell.

[b]$R_{merge} = \sum|I_j - \langle I\rangle|/\sum I_j$, where I_j is the intensity of an individual reflection and $\langle I\rangle$ is the average intensity of that reflection.

[c]R_{work} (R_{free}) = $\sum||F_o| - |F_c||/\sum|F_o|$; 5.0% of data were used for R_{free}.

Supplementary Table 4. Interactions between TCR LLL8 and HLA-A2

HLA-A2	TCR LLL8	
	Hydrogen bonds	Van der Waals contacts
α1		
E58H		D27α(1)
G62H		G94α(1)
R65H	Q96α(O) - R65H(Nη1)<br>Q96α(O) - R65H(Nη2)<br>Y48β(OH) - R65H(Nη2)	E93α(1), G94α(3), A95α(2),<br>Q96α(5), K97α(2)<br>Y48β(6)
K66H		Q31α(1), G94α(6), A95α(7)
A69H		A95α(1)<br>Y48β(1)
Q72H	Y48β(OH) - Q72H(Nε2)<br>Q50β(Oε1) - Q72H(Nε2)<br>Q50β(Nε2) - Q72H(O)	Y48β(2), Q50β(9), P55β(5)
T73H		Q50β(3), L98β(1)
α2		
A150H		D101β(3)
Q155H		Y51α(3)
A158H		S52α(2)
Y159H		Q31α(2)
T163H		Q31α(3), K67α(1)
E166H	R28α(Nη1) - Q166H(Oε2)<br>R28α(Nη2) - Q166H(Oε2)	R28α(7), K67α(2)
W167H		K67α(1)
R170H		R28α(3)

Contact residues were identified with CONTACT (**17**). Hydrogen bonds were calculated using a cut-off distance of 3.5 Å. The cut-off distance for van der Waals contacts was 4.0 Å.

Supplementary Table 5. Interactions TCR LLL8 and LLL peptide

LLL	TCR LLL8	
	Hydrogen bonds	Van der Waals contacts
L2p	Q31α(Nε2) - L2p(O)	Q31α(1)
D4p	S32α(N) - D4p(Oδ2)<br>S32α(Oγ) - D4p(Oδ2)<br>Q96α(Nε2) - D4p(O)	Q31α(9), S32α(4),<br>R92α(2), A95α(2), Q96α(3)
R5p	Q31α(Oε1) - R5p(Nη2)	Q31α(5), S32α(2), Y51α(5),<br>R92α(1), Q96α(2), G99β(1)
L6p	G99β(N) - L6p(O)	Q96α(5), D97β(1), L98β(1), G99β(6)
N7p		D97β(2)
Q8p	Q50β(Nε2) - Q8p(Oε1),<br>D97β(Oδ1) - Q8p(N)	T30β(1), Q50β(1), D97β(2)

Contact residues were identified with CONTACT (**17**). Hydrogen bonds were calculated using a cut-off distance of 3.5 Å. The cut-off distance for van der Waals contacts was 4.0 Å.

Supplementary Table 6. Computational alanine scanning to identify binding hotspot peptide and MHC residues

Mutation[1]	Chain	$\Delta\Delta G$[2]
E58A	MHC	0
Y59A	MHC	0
D61A	MHC	0.2
G62A	MHC	-0.1
E63A	MHC	0
R65A	MHC	0.2
K66A	MHC	0.5
V67A	MHC	0
A69G	MHC	0.4
H70A	MHC	0.2
Q72A	MHC	**1.9**
T73A	MHC	-0.1
H74A	MHC	0
R75A	MHC	0.1
V76A	MHC	0
K146A	MHC	0
W147A	MHC	0.3
A149G	MHC	0
A150G	MHC	0
H151A	MHC	0
V152A	MHC	0
Q155A	MHC	0.6
A158G	MHC	0.4
Y159A	MHC	0.3
T163A	MHC	-0.1
E166A	MHC	-0.1
W167A	MHC	0.2
R170A	MHC	0
L1A	peptide	0.3
L2A	peptide	0
L3A	peptide	0
D4A	peptide	**1.2**
R5A	peptide	**1.3**
L6A	peptide	0.2
N7A	peptide	**1**
Q8A	peptide	0.7
L9A	peptide	0

[1]Only peptide or MHC residues within 6 Å of LLL–HLA-A2 for the wild-type complex structure were mutated to alanine. Wild-type alanine residues were mutated to glycine.

[2]$\Delta\Delta G$ for alanine substitution, calculated by Rosetta. Values in bold are those with score ≥ 0.8 and reflect predicted binding hotspot residues for the interaction with the LLL8 TCR.

Supplementary Table 7. Computational alanine scanning to identify binding hotspot TCR residues

Mutation[1]	Chain	$\Delta\Delta G$[2]
D27A	α	0.4
R28A	α	0
S30A	α	0
Q31A	α	**1.2**
S32A	α	**0.9**
Y51A	α	**1.6**
S52A	α	-0.1
K67A	α	-0.2
R92A	α	0.4
E93A	α	0
A95G	α	**0.8**
Q96A	α	**1**
K97A	α	0.1
T30A	β	0.5
Y48A	β	0.2
Q50A	β	**0.9**
S53A	β	**0.8**
P55A	β	0.1
D56A	β	0.1
L96A	β	0
D97A	β	**2.5**
L98A	β	0.5
A100G	β	0
D101A	β	0.6

[1]Only TCR residues within 6 Å of LLL-HLA-A2 for the wild-type complex structure were mutated to alanine. Wild-type alanine residues were mutated to glycine, and glycine residues were omitted from ΔΔG calculations.

[2]$\Delta\Delta G$ for alanine substitution, calculated by Rosetta. Values in bold are those with score ≥ 0.8 and reflect predicted binding hotspot residues for the interaction with LLL–HLA-A2.
